# Effects of Creatine Monohydrate Gummies on Performance and Body Composition in Female Beach Volleyball Athletes

**DOI:** 10.3390/jfmk11010105

**Published:** 2026-03-04

**Authors:** Flavia Pereira, Scott C. Forbes, Victor Romano, Paul Christopher, Juan Carlos Santana, Jose Antonio

**Affiliations:** 1 Department of Health Sciences, Rocky Mountain University of Health Professions, Provo, UT 84606, USA; flavia.pereira@rm.edu (F.P.); victor.romano@rm.edu (V.R.); 2 Department of Exercise & Sport Science, Keiser University, West Palm Beach, FL 33409, USA; 3 Department of Physical Education Studies, Brandon University, Brandon, MB R7A6A9, Canada; forbess@brandonu.ca; 4 Gravity & Oxygen Fitness, Boca Raton, FL 33432, USA; pc@gravityandoxygen.com; 5 Institute of Human Performance, Boca Raton, FL 33432, USA; jcs@ihpfit.com; 6 Department of Health and Human Performance, NSU Florida, Davie, FL 33314, USA

**Keywords:** female athletes, ergogenic aids, randomized controlled trial, sports performance, supplements, countermovement jump, reaction time

## Abstract

**Background**: Beach volleyball is a high-intensity, intermittent sport requiring repeated explosive actions and rapid changes of direction performed on an unstable sand surface. Creatine monohydrate (CrM) supplementation has consistently been shown to enhance short-duration, high-intensity performance; however, evidence in female athletes and sport-specific contexts in beach volleyball remains limited. The purpose of this study was to examine the effects of CrM supplementation delivered in gummy form on physical performance outcomes, body composition, and reaction time in female beach volleyball athletes. **Methods**: Thirty-two female collegiate and professional beach volleyball athletes completed a 10-week randomized controlled trial and were assigned to either CrM, 5 g·day^−1^ group (*n* = 17) or control group (*n* = 15). Countermovement jump (CMJ) height, change-of-direction speed (CODS), body composition, and reaction time were assessed before and after the intervention. Outcomes were analyzed using mixed-model analyses of variance. **Results**: Significant Group × Time interactions were observed for CMJ height and CODS, with the CrM group demonstrating improvements in jump height (*p* < 0.001, ηp^2^ = 0.34) and faster change-of-direction performance (*p* = 0.009, ηp^2^ = 0.21), while the control group showed no improvement or performance declines. Significant Group × Time interactions were also observed for body fat mass (*p* = 0.024, ηp^2^ = 0.16), body fat percentage (*p* = 0.015, ηp^2^ = 0.18), and total body water (*p* = 0.038, ηp^2^ = 0.14). No significant interactions were observed for lean body mass, skeletal muscle mass, total body mass, or reaction time. **Conclusions**: CrM supplementation delivered in gummy form enhanced selected performance outcomes and helped maintain body composition in female beach volleyball athletes. These findings support creatine gummies as a practical supplementation strategy in this population.

## 1. Introduction

Beach volleyball is a high-intensity, intermittent sport characterized by repeated explosive actions such as jumping, sprinting, and rapid changes of direction performed on an unstable sand surface [1,2,3]. Competitive play requires frequent maximal or near-maximal efforts interspersed with brief recovery periods, placing substantial demands on neuromuscular performance and metabolic efficiency [4,5,6]. Vertical jump ability and change-of-direction speed are particularly important determinants of success in beach volleyball, where athletes may perform hundreds of jumps and repeated lateral movements during matches and multi-day tournaments [5]. Despite the sport’s continued growth at collegiate and professional levels, evidence-based nutritional strategies to support performance in beach volleyball, particularly among female athletes, remain limited.

Creatine monohydrate (CrM) is one of the most extensively studied ergogenic aids and has consistently been shown to enhance short-duration, high-intensity performance by increasing intramuscular creatine and phosphocreatine stores [7,8,9,10,11,12]. These adaptations support rapid adenosine triphosphate resynthesis during repeated maximal efforts, making creatine particularly relevant for activities such as jumping, sprinting, and explosive changes of direction. Position stands, and meta-analyses have demonstrated the benefits of creatine supplementation for strength and power performance across a variety of athletic populations [8,13,14,15]. However, much of this research has focused on male athletes or mixed-sex cohorts, with relatively few studies examining female athletes or sport-specific demands, such as sand-based movements.

Body composition responses to CrM supplementation remain an area of ongoing discussion. While creatine use has often been associated with increases in body mass, these changes are not consistently observed and may reflect alterations in intracellular water content rather than gains in fat-free tissue [7,16]. Studies in trained athletes report variable effects on lean body mass and fat mass, particularly when supplementation is not paired with a standardized or progressive resistance training stimulus [17,18]. In sports such as beach volleyball, where power-to-weight ratio and movement efficiency are critical, understanding whether creatine supplementation alters body composition or fluid distribution without detrimental effects is of practical importance.

In addition to its well-established role in muscular energy metabolism, creatine has been proposed to exert potential cognitive benefits, particularly under conditions of fatigue, sleep deprivation, or increased cognitive demand [19]. However, findings in healthy, well-rested athletic populations are inconsistent, with several studies reporting minimal or no effects on reaction time or sustained attention [20,21]. This divergence suggests that creatine’s cognitive effects may be context-dependent and highlights the need for sport-specific investigations that assess both physical and cognitive performance outcomes.

Importantly, the form in which creatine is consumed may influence its practical application in athletic populations. While the majority of creatine research has utilized powder formulations, alternative delivery forms such as creatine gummies have recently gained popularity, particularly among younger athletic populations. However, the physiological and performance effects of creatine delivered in gummy form remain unexplored. Emerging testing of varied gummy brands suggests that creatine content and stability may vary substantially across commercially available gummy products, underscoring the need for controlled investigations using verified formulations [22]. To date, no studies have examined the effects of creatine monohydrate delivered via gummies on performance or body composition outcomes in athletic populations, nor have any evaluated creatine supplementation in female beach volleyball athletes.

Therefore, the primary aim of this study was to examine the effects of a 10-week CrM supplementation protocol, delivered in gummy form, on countermovement jump performance, change-of-direction speed, reaction time, and body composition in female beach volleyball athletes. It was hypothesized that creatine supplementation would result in greater improvements in explosive performance outcomes and body composition, compared with a non-supplemented control group; cognitive performance responses would be more variable.

## 2. Materials and Methods

This study employed a randomized controlled trial approved by the Institutional Review Board (protocol #2025-172; approval date: 14 July 2025). The study was not prospectively registered in a public clinical trials database. A quantitative, pre-test/post-test, mixed-model design was used to examine the effects of CrM supplementation on vertical jump height, change-of-direction speed, reaction time, and body composition in female beach volleyball athletes. Participants were assigned to either a CrM supplementation group or a non-supplemented control group and followed over a 10-week intervention period.

The study included one between-subjects factor (Group: CrM vs. Control) and one within-subjects factor (Time: PRE vs. POST). This design allowed for evaluation of changes in performance and body composition outcomes over time and assessment of group-by-time interaction effects.

### 2.1. Participants & Sampling Supplementation Protocol

A total of 32 participants completed all testing sessions and were included in the final analysis. Sample size estimation, for the overall sample, was informed by a priori power analysis conducted using G*Power software (version 3.1.9.7), with countermovement jump height selected as the primary outcome variable due to its consistent reporting in the CrM literature [8]. A minimum of 30 participants were needed.

Participants were eligible if they were biological females aged 18–40 years, actively competing in an NCAA Division I or II program (https://www.ncsasports.org/beach-volleyball/colleges (accessed on 14 March 2024)) or professional beach volleyball, engaged in a minimum of 4 h·week^−1^ of volleyball training, had not consumed creatine or creatine-containing supplements within the previous six weeks, and were willing to comply with all study procedures and provide written informed consent. Participants were excluded if they had a current or chronic injury limiting full participation in training or competition, were pregnant or planning pregnancy during the study period, or had any medical or logistical factor likely to impair protocol adherence, including anticipated extended travel, major changes in diet or training, or inability to attend scheduled testing sessions.

Participants were recruited using convenience sampling from collegiate and professional female beach volleyball programs in South Florida through direct contact with coaching staffs and athletic departments, as well as targeted social media outreach. Interested athletes completed a digital screening questionnaire administered via Google Forms to determine eligibility prior to providing informed consent. Recruitment materials included flyers and social media posts containing a QR code linking to the screening questionnaire. Eligible participants were subsequently contacted by the Principal Investigator and scheduled for baseline testing.

Participants were randomized to either the CrM group or the control group using a blocked randomization procedure with a 1:1 allocation ratio. For athletes recruited from the same institution, randomization was performed within that cohort to ensure balanced group assignment, while professional athletes recruited independently were randomized without institutional stratification. Each participant was assigned a unique study identification code used for all data records and analyses. Participant identity information was stored separately from study data in a password-protected file accessible only to the Principal Investigator.

### 2.2. Supplementation Protocol

Participants assigned to the CrM group consumed 5 g of CrM gummies (Create^®^, New York, NY, USA) daily throughout the 10-week intervention period, while the control group received neither creatine nor a placebo. All participants were instructed to maintain their habitual training routines, dietary intake, and recovery practices throughout the study period. Supplement adherence and potential confounding factors were monitored through weekly questionnaires. However, dietary intake was not strictly controlled or quantified.

### 2.3. Data Collection Procedures

All testing was conducted at participating institutions’ strength and conditioning facilities or at an IRB-approved local gym under standardized conditions. Participants completed two testing sessions at baseline and post-intervention, during which body composition, reaction time, vertical jump performance, and change-of-direction speed were assessed. Participants were instructed to refrain from strenuous physical activity for at least 24 h prior to testing and to maintain their usual dietary intake on the day before and morning of each testing session.

### 2.4. Outcome Measures

Body composition was assessed using a multi-frequency bioelectrical impedance analyzer (InBody^®^ 270, InBody Co., Ltd., Seoul, Republic of Korea) to determine body mass, skeletal muscle mass, body fat mass, body fat percentage, and lean body mass, reported in SI units [23].

Lower-body explosive performance was assessed using the countermovement jump. Jump height was measured using validated jump assessment systems, including a force plate system (VALD Performance, Pty Ltd., Brisbane, QLD, Australia) at the collegiate testing site and a portable jump mat system (Jump Mat; SimpliFaster LLC, Gurnee, IL, USA) at the IRB-approved gym location. Each participant was assessed using the same device at both pre- and post-intervention testing to ensure within-subject measurement consistency. Participants completed three maximal-effort jumps with hands placed on the hips, and the highest jump height (cm) was used for analysis.

Reaction time was assessed using the Psychomotor Vigilance Task administered via Vigilance Buddy software (version 1.56), with average response time (ms) used as the primary outcome variable.

Change-of-direction speed was assessed using the 5–10–5 pro-agility shuttle test, with electronic timing gates used to record performance. Participants completed three trials per direction, and the fastest time, to the nearest 0.1 s, was retained for analysis.

### 2.5. Statistical Analysis

All statistical analyses were performed using Intellectus Statistics 360 (I360; Intellectus Statistics, LLC, Palm Harbor, FL, USA). Descriptive statistics are reported as means ± standard deviations, and statistical significance was set at α = 0.05. Mixed-model analyses of variance were conducted for each dependent variable, with Group (CrM vs. Control) as the between-subjects factor and Time (PRE vs. POST) as the within-subjects factor. Because this model accounts for baseline values and within-subject changes over time, additional independent or paired *t*-tests on descriptive tables were not performed to avoid redundancy and inflation of Type I error. Model assumptions were evaluated prior to analysis, partial eta squared (ηp^2^) was reported as a measure of effect size, and Tukey-adjusted post hoc comparisons were conducted where appropriate.

The data sets generated and analyzed during the current study are not publicly available due to ethical and privacy considerations but are available from the corresponding author upon reasonable request. Generative artificial intelligence tools were used to assist with manuscript drafting and language refinement. All scientific content, data analysis, interpretation, and conclusions were generated, reviewed, and approved by the authors.

## 3. Results

Thirty-two female beach volleyball athletes completed the study and were included in the final analyses, with 17 participants assigned to the CrM group and 15 to the control group. All participants completed pre- and post-intervention testing for countermovement jump performance, change-of-direction speed, body composition, and cognitive reaction time measures. Independent-samples *t*-tests revealed no significant baseline differences between groups for age, height, body mass, skeletal muscle mass, body fat mass, or change-of-direction speed (*p* > 0.05; Table 1). Participant characteristics are presented in Table 1. Pre- and post-intervention descriptive statistics for all outcome variables by group are presented in Table 2.

### 3.1. Countermovement Jump

**Figure 1:**
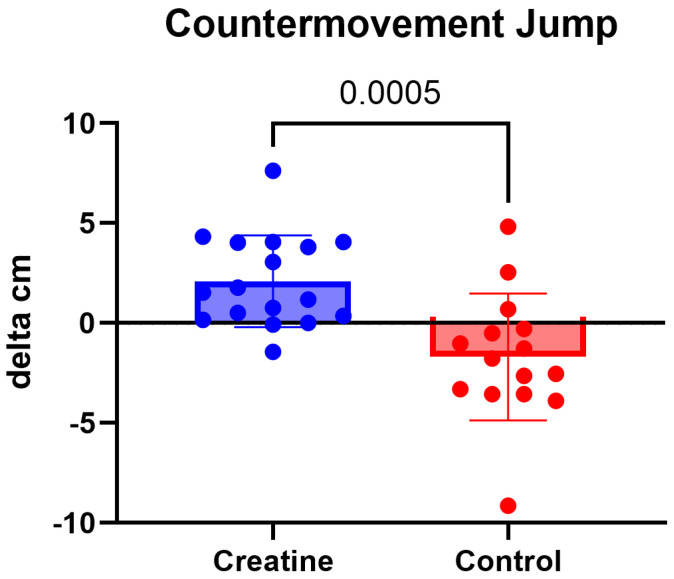
(Δ) CMJ (cm) (POST − PRE) in the CrM (+2.10 ± 2.30 cm) and control (−1.70 ± 3.18 cm) groups. Values are mean ± SD. Individual data points represent each participant.

Countermovement jump performance demonstrated a significant Group × Time interaction, F (1, 30) = 15.20, *p* < 0.001, ηp^2^ = 0.34, indicating differential changes between groups over the intervention period (Table 3; Figure 1). Post hoc analyses showed that the CrM group increased CMJ height from pre-intervention (41.8 ± 8.6 cm) to post-intervention (43.9 ± 9.0 cm), corresponding to a mean improvement of 2.10 cm (*p* = 0.004). In contrast, the control group demonstrated a decrease in CMJ height from pre-intervention (43.2 ± 8.9 cm) to post-intervention (41.5 ± 9.7 cm), representing a mean change of −1.70 cm (*p* = 0.023). No significant main effects of Group or Time were observed independent of the interaction.

### 3.2. Change of Direction Speed

**Figure 2:**
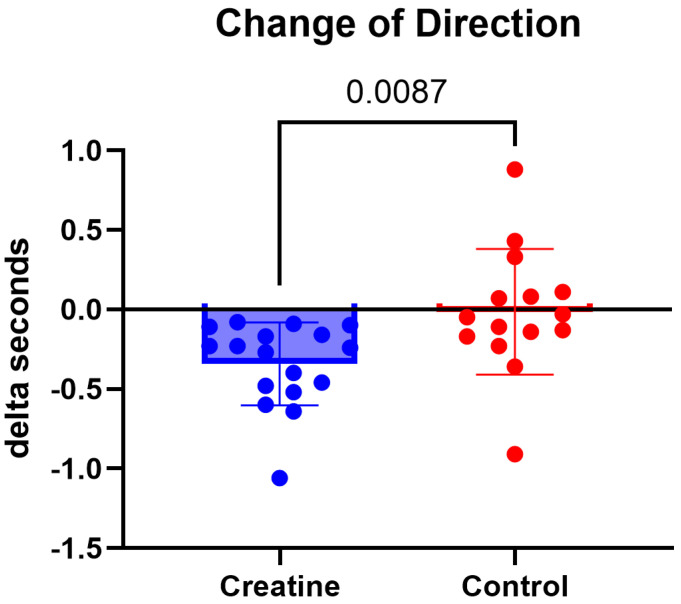
(Δ) change-of-direction speed (s) (POST − PRE) in the CrM (−0.34 ± 0.26 s) and control (−0.02 ± 0.39 s) groups. Values are mean ± SD. Individual data points represent each participant.

A significant Group × Time interaction was observed for best-performance change-of-direction speed trials, F (1, 30) = 7.88, *p* = 0.009, ηp^2^ = 0.21 (Table 3; Figure 2). The CrM group improved CODS performance from pre-intervention (6.52 ± 0.41 s) to post-intervention (6.18 ± 0.38 s), reflecting a mean reduction of 0.34 s (*p* < 0.001). The control group showed no significant change over time, with performance remaining stable from pre-intervention (6.49 ± 0.39 s) to post-intervention (6.47 ± 0.44 s; *p* = 0.858).

### 3.3. Body Composition

**Figure 3:**
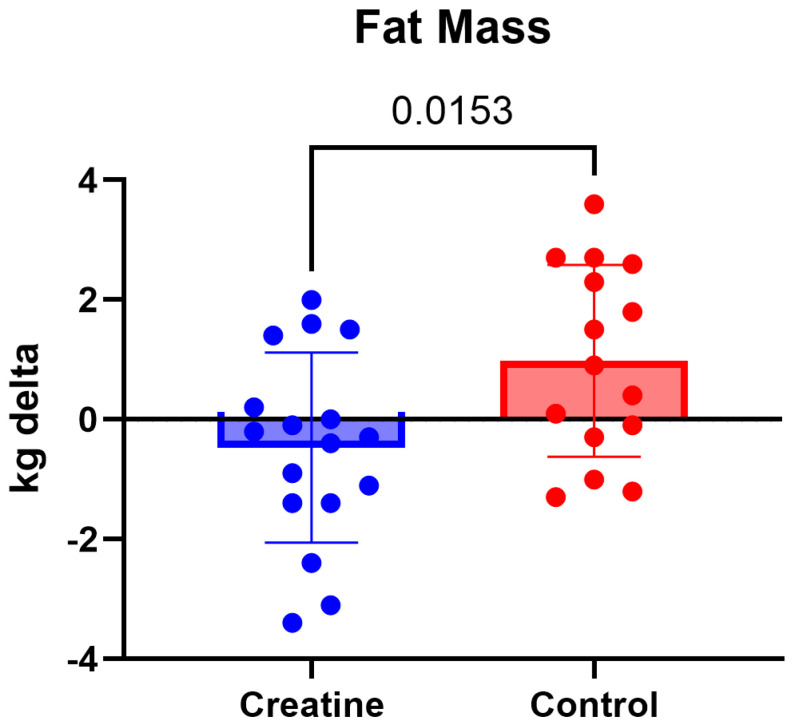
(Δ) body fat mass (kg) (POST − PRE) in the CrM −0.34 ± 1.18 kg) and control (+0.77 ± 1.46 kg) groups. Values are mean ± SD. Individual data points represent each participant.

Analysis of body composition revealed significant Group × Time interactions for both body fat mass (kg) and body fat percentage (Table 3; Figure 3). Body fat mass demonstrated a significant interaction, F (1, 30) = 5.64, *p* = 0.024, ηp^2^ = 0.16. Post hoc analyses indicated that body fat mass remained stable in the CrM group from pre-intervention (12.8 ± 3.4 kg) to post-intervention (12.9 ± 3.6 kg; *p* = 0.292), whereas the control group exhibited a significant increase from pre-intervention (13.1 ± 3.2 kg) to post-intervention (13.9 ± 3.4 kg; *p* = 0.032).

**Figure 4:**
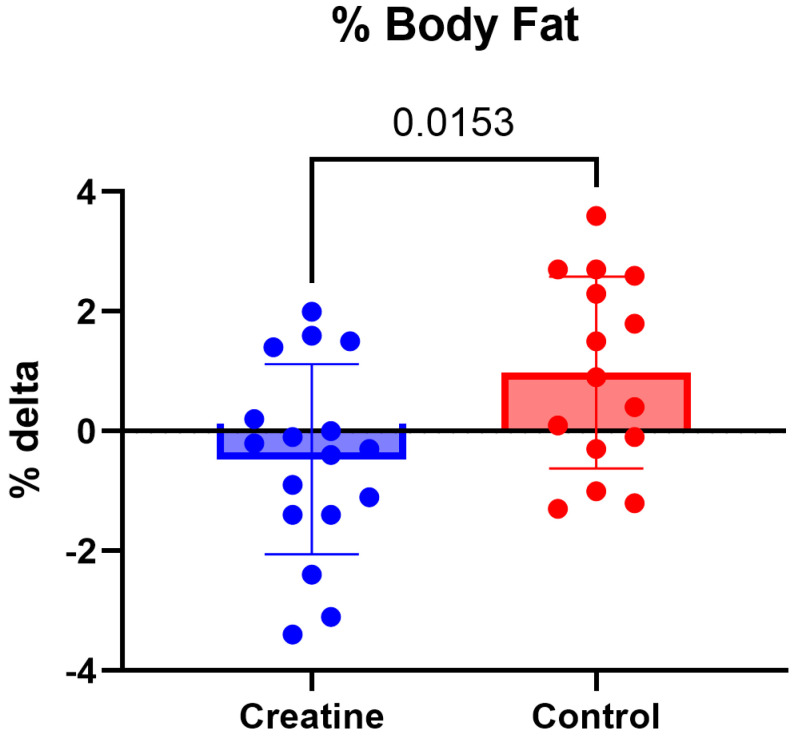
(Δ) body fat percentage (%) (POST − PRE) in the CrM (−0.47 ± 1.59%) and control (+0.98 ± 1.60%) groups. Values are mean ± SD. Individual data points represent each participant.

Similarly, body fat percentage demonstrated a significant Group × Time interaction, F (1, 30) = 6.61, *p* = 0.015, ηp^2^ = 0.18 (Table 3). Body fat percentage remained unchanged in the CrM group from pre-intervention (21.6 ± 4.1%) to post-intervention (21.7 ± 4.4%; *p* = 0.233), while the control group showed a significant increase from pre-intervention (22.0 ± 3.9%) to post-intervention (23.4 ± 4.1%; *p* = 0.024) (Figure 4).

**Figure 5:**
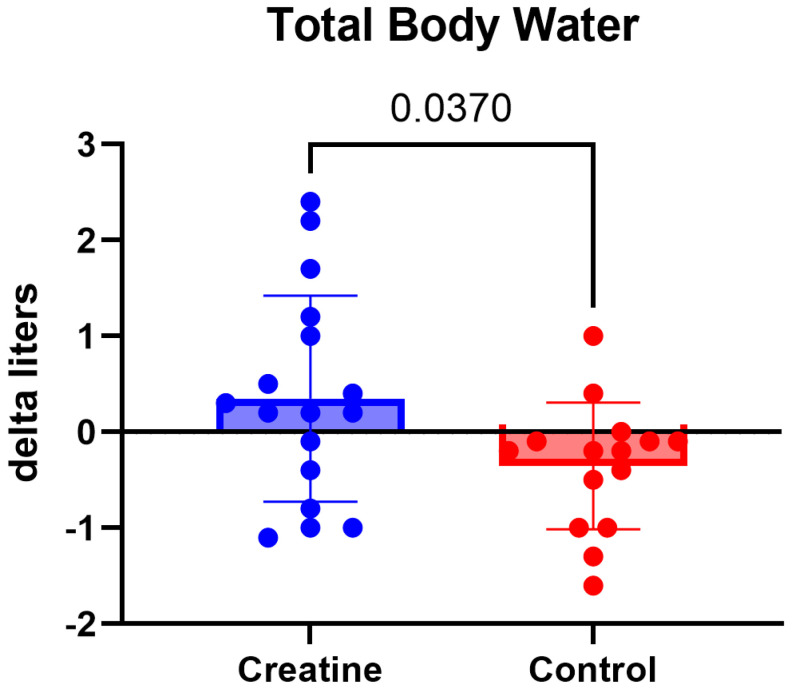
(Δ) total body water (L) (POST − PRE) in the CrM (+0.35 ± 1.08 L) and control (−0.35 ± 0.66 L) groups. Values are mean ± SD. Individual data points represent each participant.

Total body water also demonstrated a significant Group × Time interaction, F (1, 30) = 4.72, *p* = 0.038, ηp^2^ = 0.14 (Table 3). Although within-group pre–post changes were not statistically significant, the CrM group showed a small positive change in total body water, whereas the control group demonstrated a slight decrease, resulting in a divergent pattern between groups (Figure 5).

**Figure 6:**
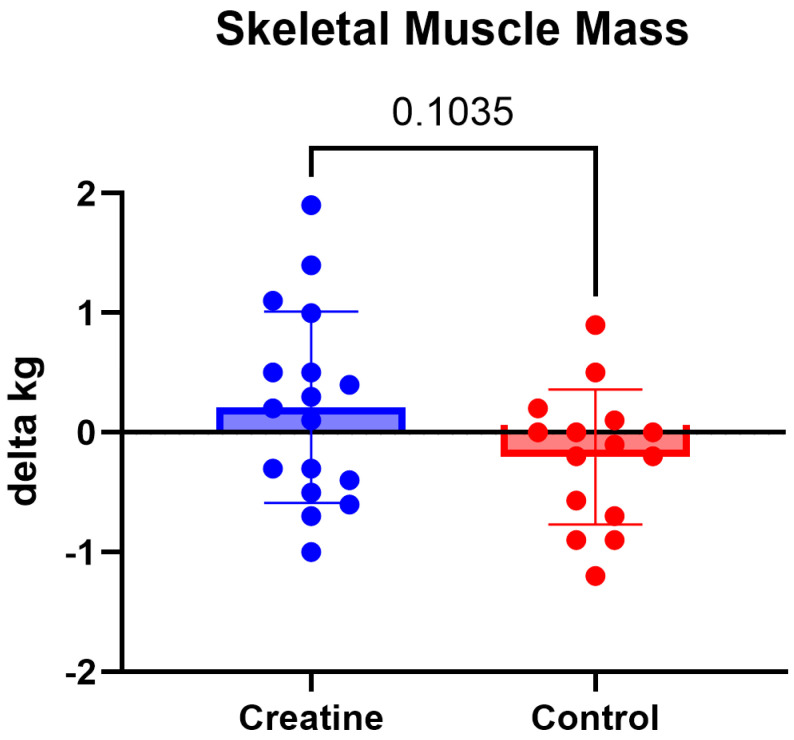
(Δ) skeletal muscle mass (kg) (POST − PRE) in the CrM (+0.21 ± 0.80 kg) and control (−0.20 ± 0.56 kg) groups. Values are mean ± SD. Individual data points represent each participant.

**Figure 7:**
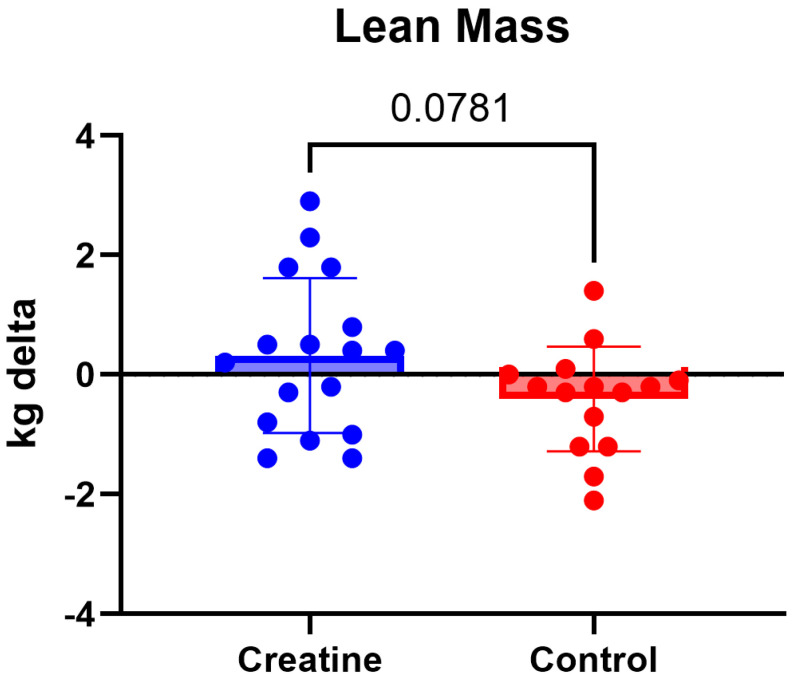
(Δ) lean body mass (kg) (POST − PRE) in the CrM (+0.32 ± 1.30 kg) and control (−0.41 ± 0.88 kg) groups. Values are mean ± SD. Individual data points represent each participant.

**Figure 8:**
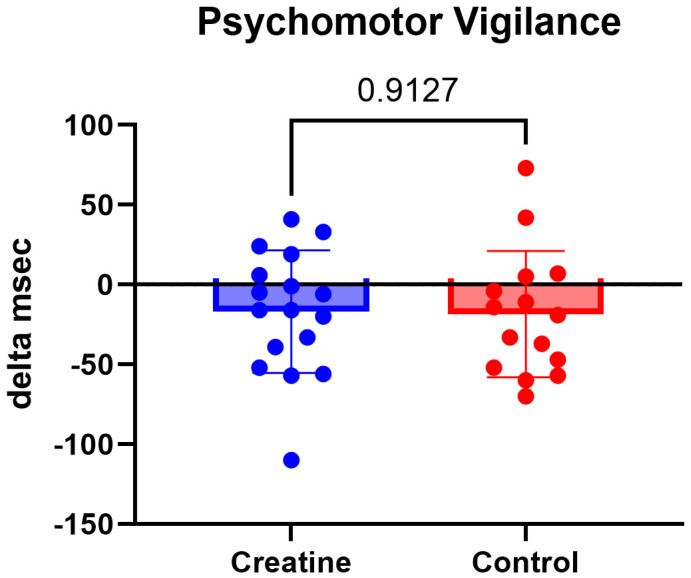
(Δ) reaction time (ms) (POST − PRE) in the CrM (−16.94 ± 38.44 ms) and control (−18.47 ± 39.52 ms) groups. Values are mean ± SD.

No significant Group × Time interactions were observed for lean body mass, skeletal muscle mass or total body mass (*p* > 0.05) (Table 3; Figure 6 and Figure 7). Lean body mass demonstrated a non-significant trend toward a differential response (F (1, 30) = 3.29, *p* = 0.078, ηp^2^ = 0.10), though changes were minimal in both groups (Table 3; Figure 8).

### 3.4. Cognitive Performance—Reaction Time

Cognitive performance, assessed via average psychomotor vigilance task response time, demonstrated a significant main effect of Time, F (1, 30) = 6.59, *p* = 0.016, ηp^2^ = 0.18, indicating faster response times from pre- to post-testing across participants. Average reaction time decreased from 285 ± 34 milliseconds (ms) to 272 ± 31 ms in the CrM group and from 289 ± 36 ms to 275 ± 33 ms in the control group. The Group × Time interaction was not significant (*p* = 0.913), indicating that improvements in reaction time did not differ between groups (Table 3; Figure 8).

## 4. Discussion

The most important finding of the present study was the significant improvement in countermovement jump (CMJ) performance observed in the creatine group, contrasted by a decline in jump performance in the control group. Vertical jump performance is a well-established indicator of lower-body explosive power and is highly relevant to beach volleyball, where repeated jumping actions are central to spiking, blocking, and offensive transitions [3]. Prior research consistently demonstrates that CrM supplementation enhances short-duration, high-intensity performance, particularly in movements reliant on the ATP-phosphocreatine energy system [7,9,10]. The present findings align with this literature and extend it to a female beach volleyball population, which remains underrepresented in creatine research.

The divergent trajectories between groups suggest that CrM supplementation may not only enhance explosive capacity but also attenuate performance decrements over a competitive training period. Given the high neuromuscular demands and cumulative fatigue associated with beach volleyball training and match play, creatine’s role in facilitating rapid phosphocreatine resynthesis and recovery between efforts may be particularly advantageous in this sport context [24].

Change-of-direction speed improved significantly following CrM supplementation. These findings are consistent with previous research demonstrating that creatine supplementation enhances performance in short-duration, high-intensity tasks requiring rapid acceleration and force production, including sprinting and change of direction-based movements, through increased phosphocreatine availability and improved ATP resynthesis [8,14,25]. The present results suggest that CrM supplementation can positively influence change-of-direction performance in female beach volleyball athletes, supporting its application in sports characterized by repeated explosive movements and high neuromuscular demands.

Emerging evidence suggests that women may begin with lower baseline intramuscular creatine concentrations compared with men, which could theoretically increase responsiveness to creatine supplementation [26]. Although baseline muscle creatine content was not measured in the present study, this framework may help contextualize the observed improvements in explosive performance following supplementation. These findings contribute to the body of literature examining creatine responses in female athletes and underscore the importance of sex-specific research that accounts for baseline creatine availability and sport-specific demands.

Body composition outcomes revealed significant group-by-time interactions for body fat mass and body fat percentage, driven primarily by increases in the control group, while values in the CrM group remained stable. These findings differ from traditional expectations that CrM supplementation leads to increases in body mass primarily through lean tissue accretion or water-related weight gain [18,25,27]. However, the absence of significant changes in skeletal muscle mass or lean body mass in either group suggests that the 10-week intervention period, despite regular participation in strength training, may have been insufficient to elicit detectable hypertrophic adaptations. This may be particularly relevant in the absence of a standardized or progressive resistance training stimulus specifically designed to maximize muscle accretion.

The stability of body fat measures in the creatine group, contrasted with increases observed in the control group, may reflect indirect effects of supplementation on training quality, recovery, or overall energy balance rather than direct effects on adipose tissue. Additionally, body composition was assessed using multi-frequency bioelectrical impedance analysis, which is sensitive to hydration status and fluid distribution [28]. Although testing conditions were standardized, subtle shifts in intracellular water associated with creatine supplementation may influence impedance-derived estimates and should therefore be interpreted with caution.

Total body water also demonstrated a significant group-by-time interaction, with the creatine group showing a small positive change and the control group a slight decrease. While within-group pre–post changes were not statistically significant, the divergent directional pattern supports existing evidence that CrM supplementation influences fluid distribution, particularly within the intracellular compartment [16,27]. This finding is consistent with prior research indicating that creatine-induced water retention is predominantly intracellular and may not necessarily translate to meaningful increases in total body mass [16].

Reaction time improved over time in both groups, with no differential effect of creatine supplementation. This finding suggests that improvements were likely attributable to familiarization with the psychomotor vigilance task or general training adaptations rather than supplementation-specific effects. Creatine’s primary ergogenic mechanisms target muscular energy metabolism, and evidence for cognitive benefits in well-rested, healthy athletes remains equivocal [21,29,30]. The task employed in the present study as well as the supplement dosage, may not have imposed sufficient cognitive or metabolic demand to reveal supplementation-related effects.

Considering recent evidence demonstrating substantial variability in creatine content across commercially available gummy formulations [22], it is noteworthy that the product used in the present study has been independently verified to contain the labeled amount of creatine monohydrate, supporting the validity of the observed performance and body composition outcomes.

### 4.1. Practical Implications

From an applied perspective, these findings support the use of CrM delivered in gummy form as a performance-enhancing supplement for female beach volleyball athletes, particularly for outcomes requiring rapid force production such as jumping and change-of-direction speed. Additionally, expectations regarding body composition changes should be tempered in the absence of structured resistance training or nutrition interventions.

### 4.2. Limitations and Future Directions

Several limitations should be acknowledged. Although the study met the required sample size based on a priori power analysis, generalizability may be limited by the relatively small and geographically concentrated sample. Training load and dietary intake were not strictly controlled, which may have influenced body composition outcomes. Additionally, the use of two different jump assessment devices across testing locations, while consistent within participants, introduces potential measurement variability.

Menstrual status was screened prior to testing and participants were rescheduled when actively menstruating to reduce acute hormonal variability; however, hormonal phase was not biochemically verified nor fully standardized between pre- and post-testing sessions. Thus, residual hormonal variation may have contributed to performance variability. Muscle creatine concentrations were not directly measured, limiting mechanistic interpretation of individual responsiveness. Additionally, the absence of a placebo control group introduces the potential for expectancy effects.

Cognitive performance outcomes should be interpreted cautiously. Reaction time is influenced by multiple factors including sleep quality, fatigue status, psychological stress, and testing familiarity. These variables were not strictly controlled, and the psychomotor vigilance task employed may not have imposed sufficient cognitive demand to detect supplementation-related differences in well-rested athletes [19,29,30].

Future research should incorporate controlled dietary and resistance training interventions and explore individual response variability to better characterize responder profiles. Further investigation into sport-specific movement patterns may also enhance understanding of how creatine supplementation interacts with complex athletic tasks in sand-based sports. Additionally, comparative studies in endurance-dominant athletes may help clarify how creatine responsiveness varies across different energetic profiles and sport demands.

## 5. Conclusions

This randomized controlled trial demonstrated that 10 weeks of creatine monohydrate supplementation in gummy form significantly improved countermovement jump performance and change-of-direction speed in female beach volleyball athletes compared with a non-supplemented control group. Supplementation was also associated with maintenance of body fat mass and percentage, whereas the control group exhibited increases over the intervention period. Total body water demonstrated a statistically significant difference between groups over time, characterized by a small increase in the creatine group and a slight decrease in the control group, without meaningful changes in total body mass. No significant effects were observed for lean body mass, skeletal muscle mass, total body mass, or reaction time.

These findings extend the creatine literature to an underrepresented athletic population and support the practical application of creatine supplementation in beach volleyball, a sand-based sport where explosive performance and power-to-weight ratio are critical [31]. Future research should further examine sex-specific responses, inter-individual variability, and the interaction between creatine supplementation and structured resistance training in beach volleyball and related court or sand-based sports.

## Figures and Tables

**Table 1:** Participant Characteristics at Baseline.

Characteristic	Creatine (*n* = 17)	Control (*n* = 15)	Baseline (*p*-Value)
Age (years)	25.7 ± 5.6	29.6 ± 9.7	0.184
Height (cm)	174.4 ± 5.1	176.5 ± 4.9	0.240
Body mass (kg, PRE)	69.8 ± 7.5	69.6 ± 5.4	0.912
BV training (h·week^−1^)	9.7 ± 5.8	9.7 ± 5.6	0.992
Weight training (h·week^−1^)	4.2 ± 1.4	3.0 ± 2.3	0.088
Total years of training	7.7 ± 4.9	10.7 ± 7.6	0.196
Other exercise (h·week^−1^)	1.1 ± 1.4	1.3 ± 1.7	0.740

Values are presented as mean ± standard deviation (SD). PRE = baseline assessment prior to intervention. Independent-samples *t*-tests were conducted to assess baseline differences between groups.

**Table 2:** Pre- and post-intervention descriptive statistics by group.

Variable	Creatine PRE	Creatine POST	Control PRE	Control POST
CMJ height (cm)	42.9 ± 8.9	45.0 ± 9.2	42.1 ± 8.8	40.4 ± 10.4
CODS (s)	5.6 ± 0.3	5.3 ± 0.3	5.8 ± 0.7	5.8 ± 0.65
Weight (kg)	69.8 ± 7.5	69.8 ± 8.1	69.6 ± 5.4	69.9 ± 5.7
Body fat mass (kg)	14.8 ± 3.8	14.4 ± 3.6	13.4 ± 3.4	14.9 ± 3.9
Body fat (%)	21.0 ± 4.2	20.5 ± 4.2	19.7 ± 4.0	20.7 ± 4.4
Lean body mass (kg)	55.8 ± 4.3	55.4 ± 4.3	55.1 ± 5.7	55.4 ± 6.3
Skeletal muscle mass (kg)	30.9 ± 3.4	31.1 ± 3.8	31.2 ± 2.5	31.0 ± 2.5
Total body water (L)	40.2 ± 4.2	40.5 ± 4.6	40.8 ± 4.2	40.5 ± 3.2
Reaction time (ms)	323.1 ± 35.2	306.2 ± 31.0	331.1 ± 22.8	313.3 ± 35.4

Values are presented as mean ± SD. PRE and POST represent measurements obtained before and after the 10-week intervention period.

**Table 3:** Mixed model ANOVA Group × Time interaction effects.

Outcome Variable	F (1, 30)	*p*-Value	ηp^2^
Countermovement jump height (cm)	15.20	<0.001	0.34
Change-of-direction speed (best side, s)	7.88	0.009	0.21
Body fat mass (kg)	5.64	0.024	0.16
Body fat percentage (%)	6.61	0.015	0.18
Lean body mass (kg)	3.29	0.078	0.10
Skeletal muscle mass (kg)	2.82	0.104	0.086
Total Body Water	4.72	0.038	0.136
Total body mass (kg)	0.51	0.479	0.017
Reaction time (ms)	0.01	0.913	<0.001

Mixed-model (2 × 2) ANOVA with Group (CrM vs. Control) and Time (PRE vs. POST) as factors. ηp^2^ = partial eta squared.

## Data Availability

The data presented in this study are not publicly available due to privacy and ethical restrictions. The dataset contains performance and body composition data from collegiate athletes collected under Institutional Review Board (IRB) approval and in accordance with school policies and agreements with team coaches. Data may be available from the corresponding author upon reasonable request and with permission from the relevant institution, subject to ethical approval.

## Abbreviations

The following abbreviations are used in this manuscript:

BV	Beach volleyball
CMJ	Countermovement jump
CODS	Change of direction speed
PVT	Psychomotor vigilance test
SMM	Skeletal muscle mass
LBM	Lean body mass
TBW	Total body water
BF%	Body fat percentage
ANOVA	Analysis of variance
η^2^p	Partial eta squared
CrM	Creatine monohydrate
ms	milliseconds
